# Diabetic Zucker rat Tibialis anterior muscle high-frequency electrical stimulation (HFES) data: Regulation of MAPKs associated proteins

**DOI:** 10.1016/j.dib.2017.11.030

**Published:** 2017-11-13

**Authors:** Gautam K. Ginjupalli, Kevin M. Rice, Anjaiah Katta, Nandini D.P.K. Manne, Ravikumar Arvapalli, Miaozong Wu, Shinichi Asano, Eric R. Blough

**Affiliations:** a Center for Diagnostic Nanosystems, Marshall University, Huntington, WV, USA; b Department of Internal Medicine, Joan C. Edwards School of Medicine, Marshall University, Huntington, WV, USA; c Biotechnology Graduate Program West Virginia State University, Institute, WV, USA; d Department of Health and Human Service, School of Kinesiology, Marshall University, Huntington, WV, USA; e Department of Public Heath, Marshall University, Huntington, WV, USA; f College of Health, Science, and Technology, University of Central Missouri, Warrensburg, MO, USA; g School of Education, Health, and Human Performance, Fairmont State University, Fairmont, WV, USA; h Department of Pharmaceutical Sciences and Research, School of Pharmacy, Marshall University, Huntington, WV, USA; i Department of Pharmacology, Physiology and Toxicology, Joan C. Edwards School of Medicine, Marshall University, Huntington, WV, USA

**Keywords:** Diabetes, Skeletal muscle, High-frequency electrical stimulation (HFES), Zucker rat, Tibialus anterior, MAPK

## Abstract

Anaerobic exercise has been advocated as a prescribed treatment for the management of diabetes: however, alterations in exercise-induced signaling remain largely unexplored in the diabetic muscle. Here, we compare the basal and the in situ contraction-induced phosphorylation of the mitogen-activated protein kinases (MAPKs) ERK 1/2, p38, and JNK in the lean and obese (fa/fa) Zucker rat tibialus anterior (TA) muscle following a single bout of contractile stimuli. This article represents data associated with prior publications from our lab (Katta et al., 2009, Katta et al., 2009, Tullgren et al., 1991) [1–3] and concurrent Data in Brief articles (Ginjupalli et al., 2017, Rice et al., 2017, Rice et al., 2017, Rice et al., 2017) [4–7].

**Specifications Table**

**Table:** 

Subject area	*Biology*
More specific subject area	*Diabetic skeletal muscle response to exercise*
Type of data	*graph, figure*
How data was acquired	*immunoblotting*
Data format	*analyzed*
Experimental factors	*A high-frequency electrical stimulation (HFES) was used to produce 10 sets of 6 contractions over a 22-minute period. Tissues were collected and protein was then isolated from tissue for western blot analysis.*
Experimental features	*TA obtained from Lean and Obese male Zucker rats were used in this experiment*
Data source location	*Huntington, WV USA*
Data accessibility	*Data is with this article and is related to articles published and in review*[1], [2], [3], [4], [5], [6], [7]

**Value of the data**

• The data presented in this brief is vital to understanding the effect of diabetes on skeletal muscle mechanotransduction.
• This data sheds light on how diabetes alters tissue response to stimuli.
• This data provides a more thorough understanding of the MAPKs involvement in exercise mediated signaling in both diabetic and non-diabetic muscle tissue.

## 1 Data

### 1.1 ERK 1/2

**Fig. 1:**
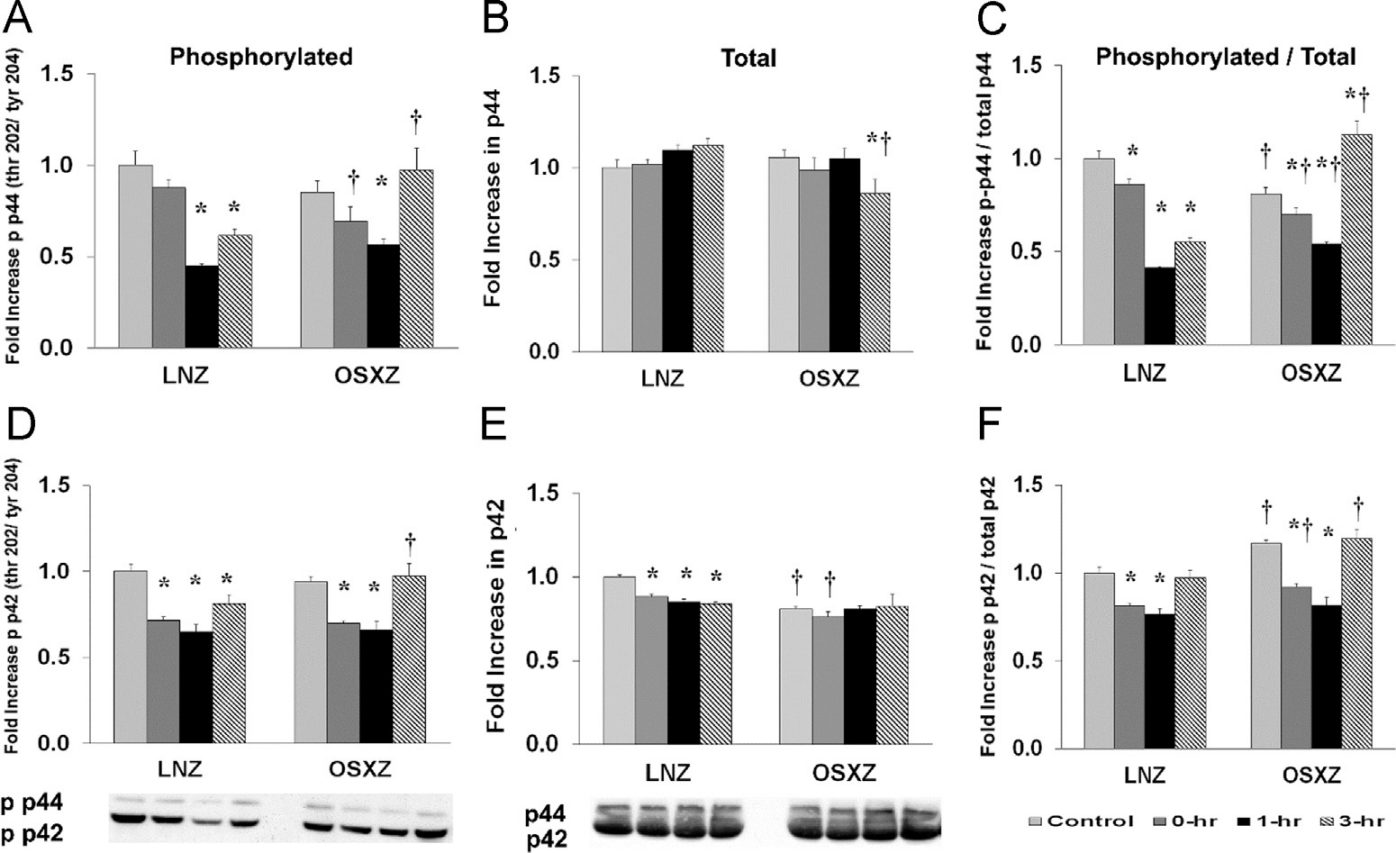
Diabetes alters HFES-induced expression and phosphorylation of p42/p44 MAPK in rat TA. The basal (control) and HFES-induced expression of p42/p44 in TA of non-diabetic lean Zucker (LNZ) and diabetic obese syndrome X Zucker (OSXZ) rats. * Significantly different from HFES TA within the same group (*p*<0.05). † Significantly different from corresponding LNZ TA (*p*<0.05). *n*=6/group.

To determine the effect of high-frequency electrical stimulation (HFES) on TA in diabetic male obese syndrome-X Zucker (OSXZ) animals and nondiabetic male lean Zucker (LNZ) animals we evaluated the expression of extracellular-signal-regulated kinase (ERK 1/2 – p44/p42). TA basal p42 content was lower (19.5±1.8%, *p*<0.05) in the OSXZ when compared to LNZ. HFES resulted in a decrease in p42 in the LNZ TA (11.8±1.5%, 15.2±1.9%, and 16.2±1.3%, at 0, 1,and 3 h, *p*<0.05) when compared to LNZ contralateral control. However HFES did not elicit a response in the OSZX TA when compared to contralateral OXSZ control. TA basal p44 content demonstrated no significant difference in the OSXZ when compared to LNZ. HFES did not elicit a change in p44 in the LNZ TA when compared to LNZ contralateral control. However HFES resulted in a decrease (19.5±7.7%, at 3 h, *p*<0.05) in the OSZX TA when compared to contralateral OXSZ control (Fig. 1).

To determine the effect of HFES on TA in OSXZ and LNZ animals we evaluated the phosphorylation of ERK 1/2 at threonine 202 and tyrosine 204 (p44/p42 thr 202/tyr 204). TA basal phosphorylation of p42 thr 202/tyr 204 was not significantly different in the OSXZ when compared to LNZ. HFES resulted in an decrease (28.4±2.1%, 35.1±4.1%, and 18.7±4.9%, at 0, 1, and 3 h, *p*<0.05) in phosphorylation of p42 thr 202/tyr 204 in the LNZ TA when compared to LNZ contralateral control. HFES resulted in a decrease (24.3±1.7% and 28.1±5.0%, at 0 and 1 h, *p*<0.05) in phosphorylation of p42 thr 202/tyr 204 in the OSXZ TA when compared to OSXZ contralateral control. TA basal phosphorylation of p44 thr 202/tyr 204 demonstrated no significant difference in the OSXZ when compared to LNZ. HFES resulted in a decrease (55.0±1.2% and 38.4±3.5%, at 1 and 3 h, *p*<0.05) in phosphorylation of p44 thr 202/tyr 204 in the LNZ TA when compared to LNZ contralateral control. HFES resulted in a decrease (28.7±2.9% at 1 h, *p*<0.05) in phosphorylation of p44 thr 202/tyr 204 in the OSXZ TA when compared to OSXZ contralateral control (Fig. 1).

To determine the effect of HFES on TA in OSXZ and LNZ animals we evaluated the ratio of phosphorylation of ERK 1/2 (p44/p42 thr 202/tyr 204) to total p42/p44. TA basal phosphorylation of p42 thr 202/tyr 204 to total p42 was higher (16.8±1.9%, *p*<0.05) in the OSXZ when compared to LNZ. HFES resulted in a decrease (18.7±1.3% and 23.6±3.2%, at 0 and 1 h, p<0.05) in phosphorylation of p42 thr 202/tyr 204 to total p42 in the LNZ TA when compared to LNZ contralateral control. HFES resulted in a decrease (24.7±1.8% and 35.4±4.6%, at 0 and 1 h, *p*<0.05) in phosphorylation of p42 thr 202/tyr 204 to total p42 in the OSXZ TA when compared to OSXZ contralateral control. TA basal phosphorylation of p44 thr 202/tyr 204 to total p44 was lower (19.1±3.2%, *p*<0.05) in the OSXZ when compared to LNZ. HFES resulted in a decrease (13.9±2.7%, 58.7±0.6%, and 44.9±2.1%, at 0, 1, and 3 hours, *p*<0.05) in phosphorylation of p44 thr 202/tyr 204 to total p44 in the LNZ TA when compared to LNZ contralateral control. HFES resulted in a decrease (11.1±3.8 and 26.7±1.1%, at 0 and 1 h, *p*<0.05) and an increase (31.9±7.1%, at 3 h, *p*<0.05) in phosphorylation of p44 thr 202/tyr 204 in the OSXZ when compared to OSXZ contralateral control (Fig. 1).

### 1.2 P38

**Fig. 2:**
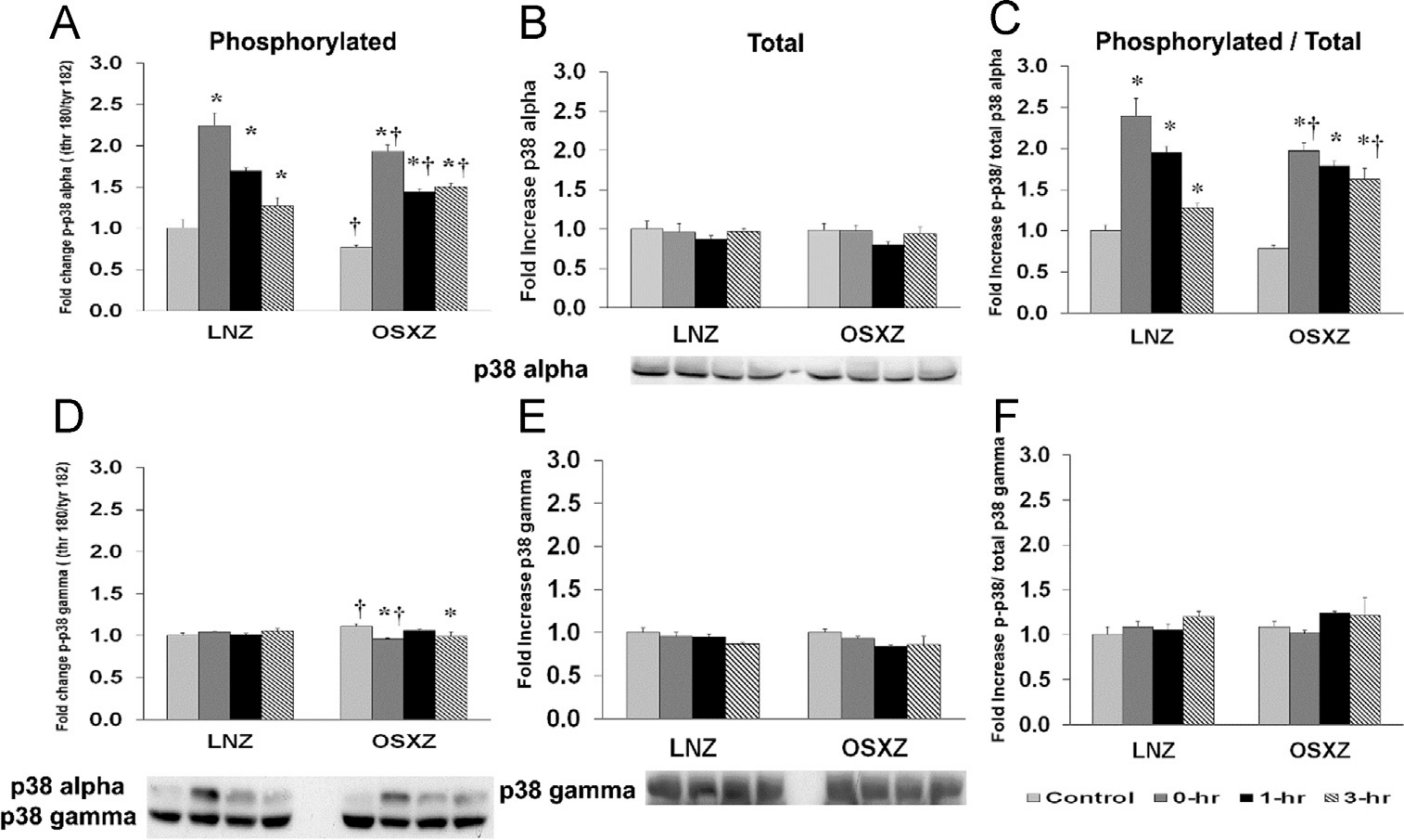
Diabetes alters HFES-induced expression and phosphorylation of p38 MAPK rat TA. The basal (control) and HFES-induced expression of p38 in TA of non-diabetic lean Zucker (LNZ) and diabetic obese syndrome X Zucker (OSXZ) rats. * Significantly different from HFES TA within the same group (*p*<0.05). † Significantly different from corresponding LNZ TA (*p*<0.05). *n*=6/group.

To determine the effect of HFES on TA in OSXZ and LNZ animals we evaluated the expression of p38 alpha and gamma. TA basal p38 alpha content demonstrated no significant difference in the OSXZ when compared to LNZ. HFES did not elicit a significant change in p38 alpha in the LNZ TA when compared to LNZ contralateral control. HFES did not elicit a response in the OSZX TA when compared to contralateral OXSZ control. TA basal p38 gamma content demonstrated no significant difference in the OSXZ when compared to LNZ. HFES did not elicit a significant change in p38 gamma in the LNZ TA when compared to LNZ contralateral control. HFES did not elicit a significant change in p38 gamma in the OSZX TA when compared to contralateral OXSZ control (Fig. 2).

To determine the effect of HFES on TA in OSXZ and LNZ animals, we evaluated the phosphorylation of p38 alpha and gamma at threonine 180 and tyrosine 182 (p38 thr 180/tyr 182). TA basal phosphorylation of p38 alpha thr 180/tyr 182 was lower (23.1±2.8%, *p*<0.05) in the OSXZ when compared to LNZ. HFES resulted in an increase (124.1±15.7%, 69.9±3.3%, and 27.1±10.1%, at 0, 1,and 3 h, *p*<0.05) in phosphorylation of p38 alpha thr 180/tyr 182 in the LNZ TA when compared to LNZ contralateral control. HFES resulted in an increase (116.4±7.8%, 67.1±4.0%, and 73.2±4.8%, at 0, 1,and 3 h, *p*<0.05) in OSXZ TA when compared to OSXZ contralateral control. TA basal phosphorylation of p38 gamma thr 180/tyr 182 was higher (10.7±1.2%, *p*<0.05) in the OSXZ when compared to LNZ. HFES did not elicit a response in phosphorylation of p38 gamma thr 180/tyr 182 in the LNZ TA when compared to LNZ contralateral control. HFES resulted in a decrease (14.3±1.2%, and 11.5±5.2%, at 0 and 3 h, *p*<0.05) in phosphorylation of p38 gamma thr 180/tyr 182 in the OSXZ TA when compared to OSXZ contralateral control (Fig. 2).

To determine the effect of HFES on TA in OSXZ and LNZ animals we evaluated the ratio of phosphorylation of p38 alpha and gamma thr 180/tyr 182 to total p38 alpha and gamma. TA basal phosphorylation of p38 alpha thr 180/tyr 182 to total p38 alpha was not significantly different in the OSXZ when compared to LNZ. HFES resulted in an increase (139.7±20.6%, 95.2±7.6%, and 28.1±5.1%, at 0, 1,and 3 h, *p*<0.05) in phosphorylation of p38 alpha thr180/tyr 182 to total p38 alpha in the LNZ TA when compared to LNZ contralateral control. HFES resulted in an increase (118.8±9.2%, 100.5±5.7%, and 84.1±12.7%, at 0, 1 and 3 h, *p*<0.05) in phosphorylation of p38 thr 180/tyr 182 to total p38 alpha in the OSXZ TA when compared to OSXZ contralateral control. TA basal phosphorylation of p38 gamma thr 180/tyr 182 to total p38 gamma demonstrated no significant difference in the OSXZ when compared to LNZ. HFES did not elicit a significant change in phosphorylation of p38 gamma thr 180/tyr 182 to total p38 gamma in the LNZ TA when compared to LNZ contralateral control. HFES did not elicit a significant change in phosphorylation of p38 gamma thr 180/tyr 182 in the OSXZ TA when compared to OSXZ contralateral control (Fig. 2).

### 1.3 JNK

**Fig. 3:**
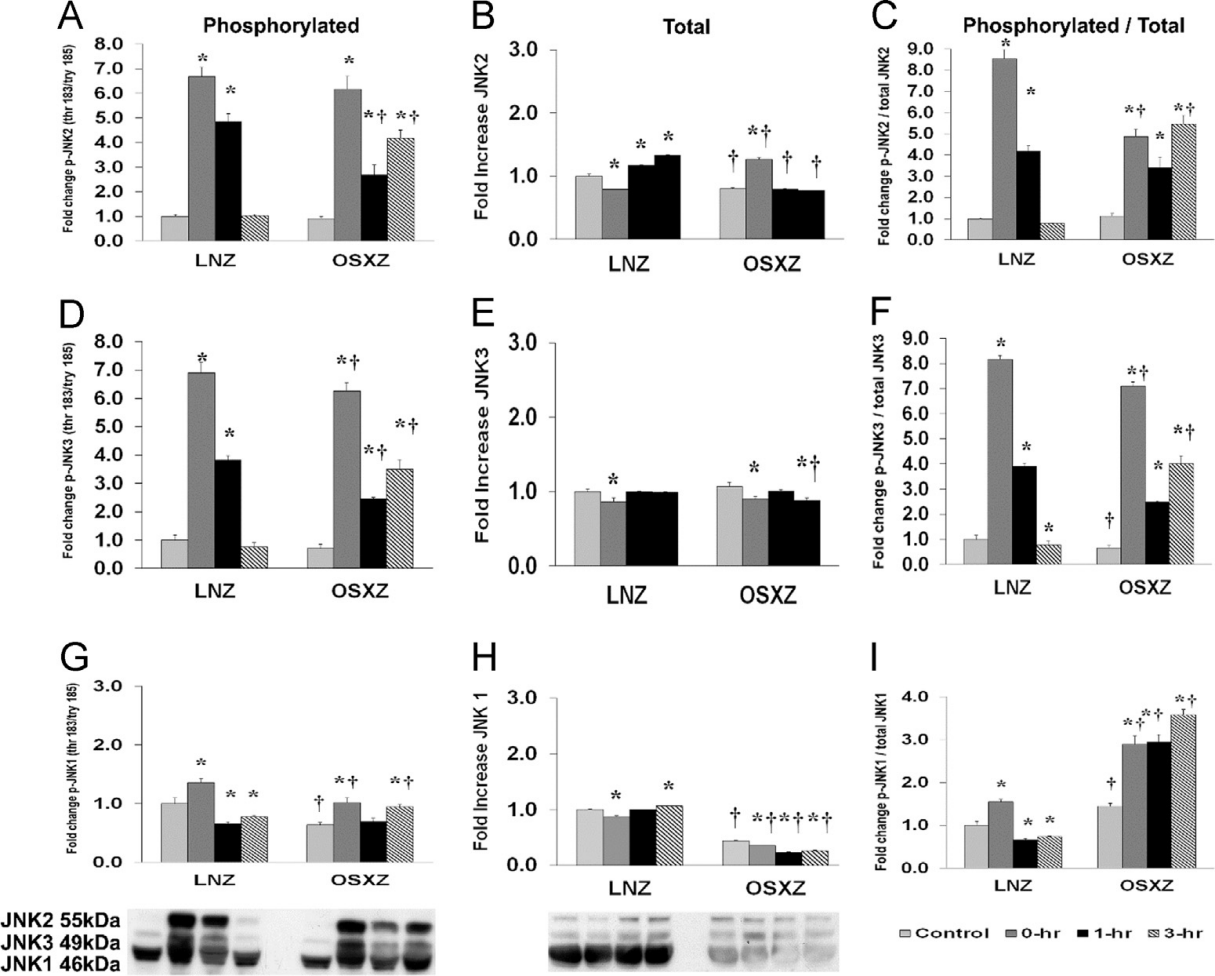
Diabetes alters HFES-induced expression and phosphorylation of JNK MAPK rat TA. The basal (control) and HFES-induced expression of JNK in TA of non-diabetic lean Zucker (LNZ) and diabetic obese syndrome X Zucker (OSXZ) rats. * Significantly different from HFES TA within the same group (*p*<0.05). † Significantly different from corresponding LNZ TA (*p*<0.05). *n*=6/group.

To determine the effect of HFES on TA in OSXZ and LNZ animals we evaluated the expression of c-Jun N-terminal kinase (JNK 1/2/3) MAPK. TA basal JNK1 was significantly lower (56.0±0.9%, *p*<0.05) in the OSXZ when compared to LNZ. HFES resulted in a decrease (12.7±1.6%, at 0 h, *p*<0.05) and an increase (6.6±0.6%, 3 h, *p*<0.05) in JNK1 in the LNZ TA when compared to LNZ contralateral control. However HFES resulted in a decrease (8.9±0.5%, 20.5±0.5%, and 17.6±0.5%, at 0, 1 and 3 h, *p*<0.05) in JNK1 in the OSXZ TA when compared to contralateral OXSZ control. TA basal JNK2 content was lower (56.0±0.9%, *p*<0.05) in the OSXZ when compared to LNZ. HFES resulted in a decrease (19.8±1.1%, at 0 h, *p*<0.05) and an increase (16.8±0.6% and 33.0±0.6%, at 1 and 3 h, *p*<0.05) in JNK2 in the LNZ TA when compared to LNZ contralateral control. HFES resulted in an increase (46.7±2.4%, at 0 h, *p*<0.05) in JNK2 in the OSXZ TA when compared to contralateral OXSZ control. TA basal JNK3 content was not significantly different in the OSXZ when compared to LNZ. HFES resulted in a decrease (13.6±5.2%, at 0 h, *p*<0.05) in JNK3 in the LNZ TA when compared to LNZ contralateral control. HFES resulted in a decrease (16.8±3.9% and 18.4±2.9%, at 0 and 3 h, *p*<0.05) in JNK3 in the OSZX TA when compared to contralateral OXSZ control (Fig. 3).

To determine the effect of HFES on TA in OSXZ and LNZ animals we evaluated the phosphorylation of JNK 1/2/3 at threonine 183 and tyrosine 185 (JNK 1/2/3 thr 180/tyr 182). TA basal phosphorylation of JNK1 thr 183/tyr 185 was lower (36.2±3.8%, *p*<0.05) in the OSXZ when compared to LNZ. HFES resulted in an increase (35.6±7.2%, at 0 h, *p*<0.05) and a decrease (33.9±2.6%, and 22.0±1.8%, at 1, and 3 h, *p*<0.05) in phosphorylation of JNK1 thr 183/tyr 185 in the LNZ TA when compared to LNZ contralateral control. HFES resulted in an increase (37.8±8.2% and 30.9±4.3%, at 0 and 3 h, *p*<0.05) in phosphorylation of JNK1 thr 1830/tyr 185 in the OSXZ TA when compared to OSXZ contralateral control. TA basal phosphorylation of JNK2 thr 183/tyr 185 was not significantly different in the OSXZ when compared to LNZ. HFES resulted in an increase (568.8±37.6% and 385.3±32.3%, at 0 and 1 h, *p*<0.05) in phosphorylation of JNK2 thr 183/tyr 185 in the LNZ TA when compared to LNZ contralateral control. HFES resulted in an increase (526.1±52.8%, 178.4±41.4%, and 328.0±32.4%, at 0, 1,and 3 h, *p*<0.05) in phosphorylation of JNK2 thr 183/tyr 185 in the OSXZ TA when compared to OSXZ contralateral control. TA basal phosphorylation of JNK3 thr 183/tyr 185 was not significantly different in the OSXZ when compared to LNZ. HFES resulted in an increase (589.8±36.5% and 283.5±14.2%, at 0 and 1 h, *p*<0.05) in phosphorylation of JNK3 thr 183/tyr 185 in the LNZ TA when compared to LNZ contralateral control. HFES resulted in an increase (555.1±29.5%, 175.0±5.6%, and 280.5±33.1%, at 0, 1,and 3 h, *p*<0.05) in phosphorylation of JNK3 thr 183/tyr 185 in the OSXZ TA when compared to OSXZ contralateral control (Fig. 2).

To determine the effect of HFES on TA in diabetic male OSXZ and LNZ animals we evaluated the ratio of phosphorylation of JNK 1/2/3 thr 183/tyr 185 to total JNK 1/2/3. TA basal phosphorylation of JNK1 thr 183/tyr 185 to total JNK1 was higher (44.7±6.1%, *p*<0.05) in the OSXZ when compared to LNZ. HFES resulted in an increase (55.1±5.8%, at 0 h, *p*<0.05) and decrease (33.7±2.3%, and 26.8±1.5%, at 1, and 3 h, *p*<0.05) in phosphorylation of JNK1 thr 183/tyr 185 to total JNK1 in the LNZ TA when compared to LNZ contralateral control. HFES resulted in an increase (144.2±19.7%, 149.2±18.0%, and 213.2±12.3%, at 0, 1, and 3 h, *p*<0.05) in phosphorylation of JNK1 thr 183/tyr 185 to total JNK1 alpha in the OSXZ TA when compared to OSXZ contralateral control. TA basal phosphorylation of JNK2 thr 183/tyr 185 to total JNK2 was not significantly different in the OSXZ when compared to LNZ. HFES resulted in an increase (753.0±41.9%, and 316.9±26.1%, at 0 and 1 h, *p*<0.05) in phosphorylation of JNK2 thr 183/tyr 185 to total JNK2 in the LNZ TA when compared to LNZ contralateral control. HFES resulted in an increase (373.7±34.2%, 227.0±50.8%, and 433.4±39.8%, at 0, 1, and 3 h, *p*<0.05) in phosphorylation of JNK2 thr 183/tyr 185 to total JNK2 alpha in the OSXZ TA when compared to OSXZ contralateral control. TA basal phosphorylation of JNK3 thr 183/tyr 185 to total JNK3 was lower (35.0±10.1%, *p*<0.05) in the OSXZ when compared to LNZ. HFES resulted in an increase (716.1±14.4% and 291.5±10.8%, at 0 and 1 h, *p*<0.05) and decrease (21.5±13.8%, at 3 h, *p*<0.05) in phosphorylation of JNK3 thr 183/tyr 185 to total JNK3 in the LNZ TA when compared to LNZ contralateral control. HFES resulted in an increase (643.5±17.4%, 183.8±3.8%, and 336.8±29.7%, at 0, 1, and 3 h, *p*<0.05) in phosphorylation of JNK3 thr 183/tyr 185 to total JNK3 alpha in the OSXZ TA when compared to OSXZ contralateral control (Fig. 3).

## 2 Experimental design, materials and methods

### 2.1 Animals

All procedures were conducted as described elsewhere [1], [2], [3], [4], [5], [6], [7] in strict accordance with the Guide for the Care and Use of Laboratory Animals as approved by the Council of the American Physiological Society and the Animal Use Review Board of Marshall University. Young (10 week, *n*=12) male lean Zucker (non-diabetic) (LNZ) and young (10 week, *n*=12) male obese syndrome-X Zucker (diabetic) (OSXZ) rats were obtained from the Charles River Laboratories and barrier housed one per cage in an AAALAC approved vivarium. Housing conditions consisted of a 12H:12H dark-light cycle and the temperature was maintained at 22±2 °C. Animals were provided food and water *ad libitum*. Rats were allowed to recover from shipment for at least two weeks before the commencement of experimentation during which time the animals were carefully observed and weighed weekly.

### 2.2 Materials

Anti-Erk1/2 (p42/p44) (#9102), phospho-Erk1/2 (P42/p44) Thr202/Tyr204 (#9106), p38 (#9212), phospho-p38 Thr180/Tyr182 (#9216), SAPK/Jnk (#9252), phospho-SAPK /JnkThr183/Tyr185 (#9251), Mouse IgG, and Rabbit IgG antibodies were purchased from Cell Signaling Technology (Beverly, MA). Enhanced chemiluminescence (ECL) western blotting detection reagent was from Amersham Biosciences (Piscataway, NJ). Precast 10% and 15% SDS-PAGE gels were purchased from Lonza (Rockland, ME). Enhanced chemiluminescence (ECL) western blotting detection reagent was purchased from Amersham Biosciences (Piscataway, NJ). Restore western blot stripping buffer was obtained from Thermo scientific (Rockford, IL). All other chemicals were from Sigma (St. Louis, MO).

### 2.3 Contractile stimulation of skeletal muscles

The high-frequency electrical stimulation (HFES) model has been previously described [8] and was chosen on the basis of its efficacy in stimulating protein translation and muscle hypertrophy in vivo [9]. The HFES model used in the present study produced 10 sets of 6 contractions with an overall protocol time of 22 min. Animals were killed by a lethal dose of sodium pentobarbital at baseline, immediately following, 1 h or 3 h (*n*=6 normal, *n*=6 diabetic for 0, 1, and 3 h) after HFES. Once excised, muscles were blotted dry, trimmed of visible fat and tendon projections, weighed, immediately frozen in liquid nitrogen, and stored at −80 °C.

### 2.4 Immunoblot analysis

Samples were prepared and immunoblotting performed as outlines elsewhere by Rice *et. al.* [1], [2], [3], [4], [5], [6], [7].

### 2.5 Data analysis

Data were analyzed using Sigma Stat 12.0 statistical software and the results are presented as mean±SEM. Two-way ANOVA followed by the Student-Newman-Keuls post-hoc test was conducted to determine differences between groups. The level of significance accepted *a priori* was<0.05.
